# Vector competence of Italian *Aedes albopictus* populations for the chikungunya virus (E1-226V)

**DOI:** 10.1371/journal.pntd.0006435

**Published:** 2018-04-19

**Authors:** Francesco Severini, Daniela Boccolini, Claudia Fortuna, Marco Di Luca, Luciano Toma, Antonello Amendola, Eleonora Benedetti, Giada Minelli, Roberto Romi, Giulietta Venturi, Giovanni Rezza, Maria Elena Remoli

**Affiliations:** 1 Unit of Vector-borne Diseases DMI Department, Istituto Superiore di Sanità, Rome, Italy; 2 Unit of Arbo, Hanta and Emerging Viruses, DMI Department, Istituto Superiore di Sanità, Rome, Italy; 3 Technical Scientific Statistics Service of the Italian National Institute of Health, Istituto Superiore di Sanità, Rome, Italy; INDEPENDENT RESEARCHER, UNITED STATES

## Abstract

**Background:**

Chikungunya virus (CHIKV) is an emerging arbovirus, belonging to the Togaviridae family, *Alphavirus* genus, transmitted by *Aedes* spp. mosquitoes. Since 2007, two different CHIKV strains (E1-226A and E1-226V) have been responsible for outbreaks in European countries, including Italy, sustained by *Ae*. *albopictus* mosquitoes.

**Findings:**

In this study, we assessed the susceptibility to the CHIKV E1-226V, strain responsible for the Italian 2007 outbreak, of eight *Ae*. *albopictus* populations collected in Northern, Central, Southern, and Island Italy, by experimental infections. Vector competence was evaluated by estimating infection, dissemination, and transmission rates (IR, DR, TR), through detection of the virus in the bodies, legs plus wings, and saliva, respectively. Additionally, vertical transmission was evaluated by the detection of the virus in the offspring. The results of our study demonstrated that the Italian populations of *Ae*. *albopictus* tested were susceptible to CHIKV infection, and can disseminate the virus outside the midgut barrier with high values of IR and DR. Viral infectious RNA was detected in the saliva of three populations from Central, Southern, and Island Italy, also tested for TR and population transmission rate (PTR) values. No progeny of the first and second gonotrophic cycle were positive for CHIKV.

**Conclusions:**

This study strongly confirms the role of *Ae*. *albopictus* as a potential CHIKV vector in Italy. This may represent a threat, especially considering both the high density of this species, which is widespread throughout the country, and the increasing number of cases of imported arboviruses.

## Author summary

*Aedes albopictus* is a proven vector of more than 20 different arboviruses and, as demonstrated by experimental infections, is an efficient vector of chikungunya virus (CHIKV) in several countries. In Italy this widespread species caused the first outbreak of CHIKV in Europe in 2007 (Emilia-Romagna region) and recently, after 10 years, two different outbreaks in the Central and Southern part of the country (Latium and Calabria regions) were reported. Symptoms of CHIKV are arthralgia and joint pain, skin rash, primarily of the trunk and limbs, but also commonly fever and myalgia. Complications of this disease are rare, but long-term sequelae often occur in a considerable number of patients in particular, in children, the elderly, and patients with chronic diseases. The recent circulation of CHIKV in Italy has highlighted the importance of investigating vector competence of Italian populations of *Ae*. *albopictus* from different areas. In this study, we experimentally infected several mosquito populations in order to assess the real risk of CHIKV epidemics in temperate zones and support efficient surveillance and control strategies.

## Introduction

Chikungunya virus (CHIKV) is a zoonotic arthropod-borne virus (Togaviridae family, *Alphavirus* genus), historically endemic in Africa. Since its first isolation during an outbreak in Tanzania in 1952, several epidemics in African and Asian continents were reported. The re-emergence of CHIKV was unpredictable, with intervals from 7 to 20 years between consecutive epidemics [1–5].

In 2004, CHIKV re-emerged, with an explosive onset in Kenya, spreading to the Comoros and the La Réunion islands and, in early 2005, to other islands in the South-West Indian Ocean. These events were followed by an epidemic in the Indian subcontinent in 2005/2006. In only 10 years, CHIKV spread across all five continents, from the Indian Ocean region to Asia, to Mediterranean Europe and Central America causing a series of large outbreaks [6–9].

In 2007, for the first time, CHIKV reached the temperate climate countries of Europe, causing more than 200 autochthonous cases in North-Eastern Italy [10]. In 2010 and 2014 local transmission events were reported also in the South-East of France, with two and twelve cases respectively [11, 12], and one autochthonous case was reported in 2015 in the South-East of Spain [13]. More recently in August 2017, other locally-acquired CHIKV infections, were reported in South-East France (Le Cannet-des-Maures, Var Department), and in three municipalities in Central and South Italy (Anzio and Rome, on Tyrrhenian coast, Latium region, and Guardavalle on the Ionic coast of Calabria region) [14–17]. These autochthonous CHIK outbreaks clearly point out the high vulnerability of Europe to the transmission of tropical arboviruses.

In urbanized areas, CHIKV transmission is sustained by the anthropophilic *Aedes* species, such as *Ae*. *aegypti* and *Ae*. *albopictus* (known as the tiger mosquito) able to cause large urban epidemics. In this context, humans act as amplifier hosts capable of developing high viremia (e.g. 10^8−9^ RNA copies/mL) [18], thus infecting other mosquitoes and contributing to the spread of the virus.

To date, three virulent CHIKV genotypes have been identified: West African, Asian, and Eastern-Central-South African (ECSA) [19]. During the Réunion islands outbreak, the emergence of an *Ae*. *albopictus*-adaptive mutation (E1-226V) in the Indian Ocean Lineage-IOL strains (ECSA genotype), provided a fitness increase of CHIKV with a shorter extrinsic incubation period (EIP) in the *Ae*. *albopictus* vector, which was widespread on the islands [18, 20–24]. This adapted viral genomic variant was involved in the outbreaks occurring in North-Eastern Italy in 2007 and in the South-Eastern France in 2014 and 2017 where *Ae*. *albopictus* is widespread [10, 16, 25]. Thus, although *Ae*. *aegypti* was widely recognized as the main urban vector of CHIKV in tropical areas, *Ae*. *albopictus* is considered able to transmit CHIKV in temperate climate areas too. The presence of field-collected mosquitoes positive to the RNA virus highlighted the role of *Ae*. *albopictus* as CHIKV vector during the European outbreaks [26, 27]. Moreover, experimental infection confirmed a high susceptibility of local European *Ae*. *albopictus* populations to the mutated ECSA CHIKV strain (E1-226V) [28–32].

Although the virus recently detected from the French index case is carrying the E1-226V mutation [16], the strain responsible for the ongoing outbreaks in Central and Southern Italy, as well as the viral strain detected in France in 2010, are not carrying this mutation [12, 15, 33].

This repeated circulation of both CHIKV strains in Europe has emphasized the importance of evaluating the vector competence of *Ae*. *albopictus* from different areas in order to assess the real risk of CHIKV epidemics in temperate zones and to support efficient surveillance and control strategies.

This study aims to experimentally evaluate the vector competence of *Ae*. *albopictus* and to assess potential susceptibility for CHIKV (E1-226V) among mosquito populations, in particular of Central, Southern, and Island Italy. In addition, the mosquito progeny from both the first (FGC) and the second (SGC) gonotrophic cycle were analyzed in order to assess the possible virus overwintering.

## Methods

### Ethics statement

This study was carried out in accordance with the recommendations of the Animal Experimentation protocol (Decree no. 116/92, European Directive 86/609/EEC). In accordance with this legislation the presence and approval of an Ethic Committee is not required; however, at the Istituto Superiore di Sanità (Rome, Italy), the veterinarians of the Service for Biotechnology and Animal Welfare, performed the functions of local IACUCs. Blood was collected from the ear vein of the rabbit according to the European legislation for the care and the use of laboratory animals. Pig intestine epithelium, used for the membrane feeding system, is a commercially available product [34].

### Mosquitoes

**Fig 1:**
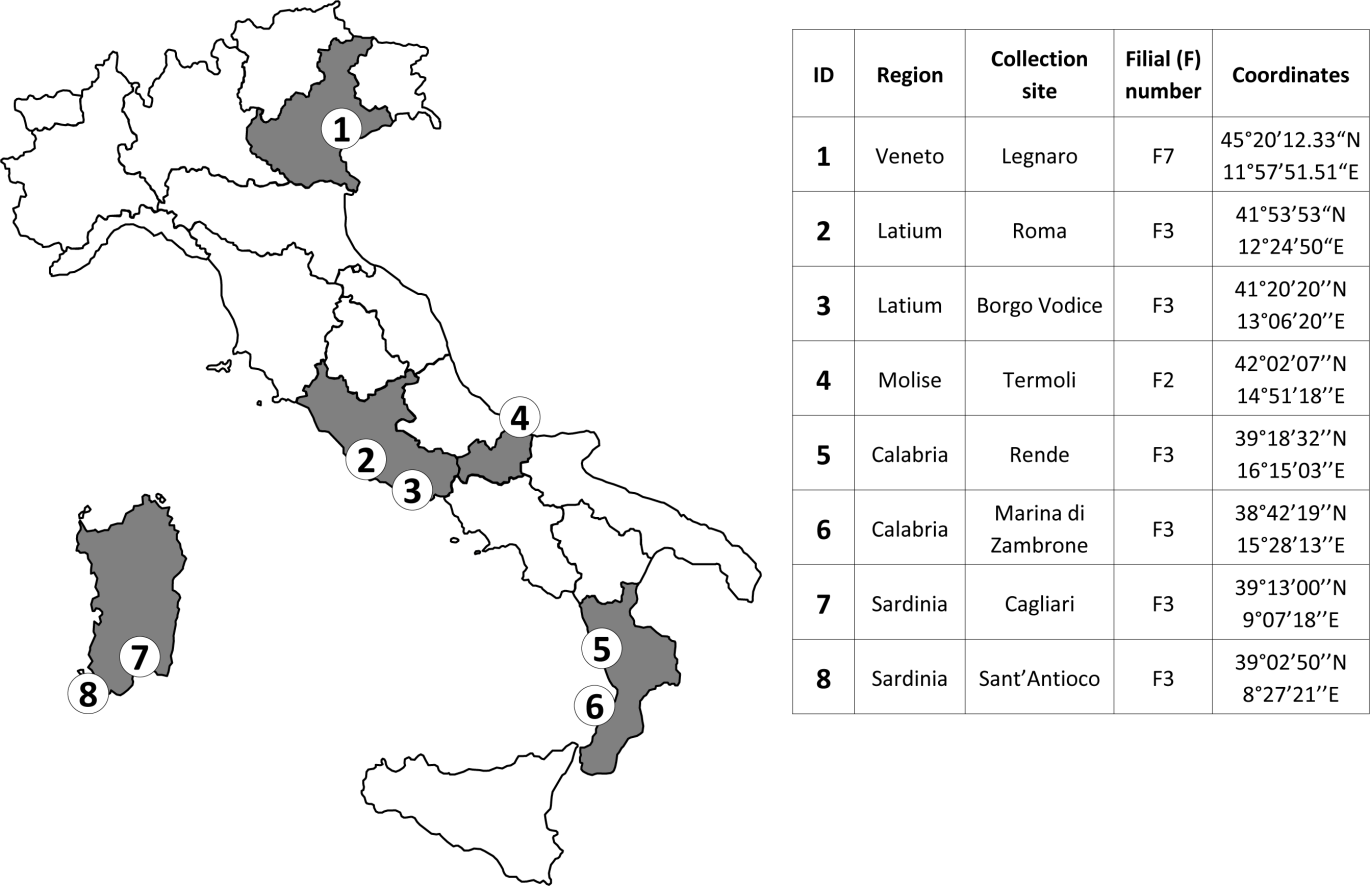
Description of *Aedes albopictus* populations. Locations and filial generations (F) of the Italian *Ae*. *albopictus* populations experimentally infected with CHIKV.

For the experimental infections, eight Italian *Ae*. *albopictus* populations, were used: one from Northern Italy: Legnaro (Padua province, Veneto region); three from Central Italy: Rome (Latium region); Borgo Vodice (Latina province, Latium region); Termoli (Campobasso province, Molise region); four from Southern Italy: Rende (Cosenza province, Calabria region); Marina di Zambrone (Vibo Valentia province, Calabria region); Cagliari (Sardinia region) and Sant’Antioco (Carbonia-Iglesias province, Sardinia region). Collection sites of tested mosquito populations are reported in Fig 1.

For each population, about 800–1000 adult mosquitoes, originated from field-collections of eggs and larvae in the 2015–2016 summer season, were used to establish the laboratory colonies and were reared for several filial generations (as shown in Fig 1) in the Insectarium of the Istituto Superiore di Sanità before experimental infection. Larvae and adults were reared and maintained following a standardized procedure [35], mosquitoes were held in a climatic chamber maintained at 27±1°C, 70% relative humidity, and a 14h:10h light-dark cycle. Larvae were reared in a 0.3% sodium chloride solution and fed with dry cat food (Royal Canin srl, 20151 Milan, Italy); emerged adults were maintained in cages and supplied with a 10% sucrose solution. To ensure egg laying, mosquito females were provided a rabbit blood meal by membrane feeding apparatus, consisting of a pig intestine membrane covering the base of a glass feeder (Vetro Scientifica srl, 00185 Rome, Italy) containing the blood.

For experimental infection 7-day-old mosquito females of the eight Italian *Ae*. *albopictus* populations were used.

All populations were tested to exclude the presence of CHIKV and dengue virus. About 100 mosquitoes of each population were pooled (20 individuals), according to the geographic origin and sex, and analyzed by using quantitative Real Time PCR (qRT-PCR).

### Experimental oral infections and specimen collection

For the experimental infections, CHIKV strain CHIKV/ISS-2007/patient G.P./M2V2, isolated on VERO cells from the serum of a patient from Emilia Romagna outbreak in 2007 [10], was used. The CHIKV stocks were obtained by propagation on VERO cells and then stored at -80°C in aliquots until processed. The viral titer used for experimental infection of CHIKV frozen stock was 6.8 log_10_ Plaque Forming Units/mL (PFU/mL) obtained by plaque assay on VERO cells.

Experimental infections of mosquito populations were performed in BSL-3 cabinet using an infectious blood meal, composed of 2/3 rabbit blood with EDTA (Ethylenediaminetetraacetic acid, Sigma-Aldrich Corp, Rockville, MD, USA) and 1/3 viral seed, with a final concentration of 6.3 log_10_ PFU/mL. Female mosquitoes were allowed to feed for 60 min through a pig intestine membrane covering the base of a glass feeder containing the blood-virus mixture maintained at 37°C by a warm water circulation system. After the infectious blood meal, fully engorged females were transferred into new cages and maintained in a climatic chamber for 12 days, at the same insectarium conditions as described earlier.

To determine if virus was present in the body (head, thorax, and abdomen) or legs plus wings of the tested mosquitoes, from three to nine specimens of each population were dissected at days 0, 2, 3, 7, and 12 post exposure (d.p.e.).

The length of viral EIP, and the trend of viral particles in the saliva samples of potentially infected females, were evaluated by collecting specimens at all d.p.e., as reported above, from three *Ae*. *albopictus* populations from Central, Southern, and Island Italy (Borgo Vodice, Rende, and Sant’Antioco, respectively). Briefly, after dissection of the legs and wings from the body, mosquitoes were forced to salivate and the proboscis was inserted into a quartz capillary filled with 3 μL of fetal bovine serum (FBS, Sigma-Aldrich, St. Louis). One microliter of 1% pilocarpine (Sigma-Aldrich, St Louis, MO) [30] was applied on the thorax. After 30 min, the medium containing the saliva was expelled into a 1.5-mL tube containing 500 μL of Mosquito Diluent (MD) buffer (Phosphate Buffer Saline, 20% heat-inactivated FBS, 1% penicillin/streptomycin/amphotericin B mix; Invitrogen, GIBCO).

Bodies, legs plus wings, and saliva specimens were stored at -80°C until processed [35–37].

To detect a potential vertical transmission of the CHIKV, a sample of potentially infected females was allowed to lay eggs. Larvae from the FGC were reared up to adulthood in the climatic chamber. Samples of adults (grouped by geographic origin and sex) were obtained from the early (4 d.p.e.) and late (7 d.p.e.) ovipositions. At 12 d.p.e. an uninfected second blood meal was provided for the remaining females of the Borgo Vodice population and offspring from the SGC (ovipositions of the 4 and 7 days after the uninfected meal) were also reared and the adults collected. All samples from FGC and SGC were stored at -80°C and processed as pools of 5–30 specimens.

### Mosquitoes’ body and legs plus wings processing and viral RNA extraction

For each mosquito, body and legs plus wings were cold homogenized separately, suspended in 1 mL and 0.8 mL of MD buffer, respectively, and centrifuged at 3000 x g for 30 min at 4°C. The supernatants were aliquoted and, together with mosquito’s saliva samples, were used for RNA extraction by using the QIAamp viral RNA kit in accordance with the manufacturer’s protocol (Qiagen Inc., Valencia, CA, USA).

### Viral titration by qRT-PCR

CHIKV titer of infected mosquitoes was evaluated by qRT-PCR, performed by using CHIKV TaqMan primers and probe [10]. Quantification of CHIKV in RNA samples was obtained comparing the crossing points of the values of the standard curve obtained from 10-fold serial dilutions of CHIKV stocks with estimated concentration by titration on VERO cells [35–37]. These values were expressed as plaque forming unit equivalents (PFUeq).

### Virus viability in saliva samples

Viral isolation was carried out as described by Verani et al. [38]. Briefly, 100 μL of the supernatant fluid of saliva were seeded on a confluent VERO cells monolayer. After 1 hour of incubation at 37°C, 2 mL of medium, consisting of Dulbecco’s MEM, 2% FBS, 1% antibiotic-antimycotic mix (Invitrogen, Gibco), was added. VERO cell cultures were examined daily for 14 days for cytopathic effect (CPE).

### Susceptibility indexes

Vector susceptibility was evaluated by analyzing the following indexes: i) the infection rate (IR) calculated as the number of CHIKV positive bodies with respect to the total number of fed females; ii) the dissemination rate (DR) calculated as the number of specimens with CHIKV-positive legs plus wings among the number of specimens with CHIKV-positive bodies; iii) the transmission rate (TR) defined as the number of mosquitoes with CHIKV-positive saliva among the number of specimens with CHIKV-positive bodies. The potential vector competence was expressed as population transmission rate (PTR), calculated as the number of specimens with CHIKV-positive saliva with respect to the total number of fed mosquitoes [35–37, 39, 40].

### Statistical analysis

The non-parametric Kruskal-Wallis test was used to compare the mean titer values in body and legs plus wings among all the mosquito populations tested. To evaluate trends in viral replication in bodies, legs plus wings, and saliva over time (expressed in d.p.e.), *nptrend* (nonparametric test), developed by Cuzick [41], was used. The values of TR and PTR among Borgo Vodice, Rende, and Sant’Antioco were compared using Chi-squared test (or Fisher-Yates test). Significant difference was established when *p*-values were lower than 0.05. Data analyses were carried out with Stata 13 software (StartCorp LP, Texas, USA).

## Results

### CHIKV replication in body and legs plus wings

Initially the study was performed to assess the susceptibility to infection and dissemination of CHIKV in eight Italian *Ae*. *albopictus* populations representative of the whole country. As shown in Table 1 all *Ae*. *albopictus* bodies analyzed at 0 d.p.e. showed qRT-PCR positive results with mean viral titers around 3–4 log_10_ PFUeq/mL. The analysis of the mosquito bodies exhibited an increase of mean viral titer from 0 to 7 d.p.e. showing that mosquitoes tested were infected and able to permit CHIKV replication in their body. Legnaro, Rome, Termoli, Rende, Marina di Zambrone, and Sant’Antioco reached the peak at 2 d.p.e. with values of 5.1±0.6, 5.5±0.5, 5.1±0.6, 5.5±0.3, 5.1±nd, and 4.8±0.3 log_10_ PFUeq/mL, respectively. Viral RNAs recovered from Borgo Vodice and Cagliari after CHIKV infection showed mean values approximately constant from 2 to 7 d.p.e. even if an increase of the mean titer was found at 7 d.p.e. (4.9±1.0 and 5.4±0.7 log_10_ PFUeq/mL respectively). Moreover, in all mosquitoes processed on the 12 d.p.e. lower RNA titers were detected if compared with 2 d.p.e., suggesting a decreasing of the viral replication. The trend of the mean viral titers for all eight populations were comparable and no statistically significant differences were observed (Kruskal Wallis test *p =* 0.825; *nptrend* values ranging from 0.259 to 0.791).

**Table 1:** Values of IR, DR and the mean CHIKV (E1-226V) titers (expressed in log_10_ PFUeq/mL) in fed females of Italian *Aedes albopictus* populations.

d.p.e.		Legnaro		Rome		Borgo Vodice		Termoli		Rende		Marina di Zambrone		Cagliari		Sant’Antioco	
		IR *[n]*^*a*^ (m.t. ± SD)	DR *[n]*^*b*^ (m.t. ± SD)	IR (m.t. ± SD)	DR (m.t. ± SD)	IR (m.t. ± SD)	DR (m.t. ± SD)	IR (m.t. ± SD)	DR (m.t. ± SD)	IR (m.t. ± SD)	DR (m.t. ± SD)	IR (m.t. ± SD)	DR (m.t. ± SD)	IR (m.t. ± SD)	DR (m.t. ± SD)	IR (m.t. ± SD)	DR (m.t. ± SD)
**0**		100 *[4]* (3.9 ± 0.3)	_	100 *[4]* (3.8 ± 0.3)	_	100 *[4]* (4.0 ± 0.1)	_	100 *[4]* (3.8 ± 0.2)	_	100 *[4]* (3.7 ± 0.2)	_	100 *[4]* (3.7 ± 0.1)	_	100 *[5]* (3.9 ± 0.1)	_	100 *[4]* (4.0 ± 0.3)	_
**2**		100 *[3]* (5.1 ± 0.6)	0 *[3]* (0)	100 *[4]* (5. 5 ± 0.5)	0 *[4]* (0)	100 *[4]* (4. 9 ± 0.9)	100 *[4]* (3.0 ± 0.5)	100 *[3]* (5.1 ± 0.6)	100 *[3]* (3.6± 0.4)	100 *[3]* (5.5 ± 0.3)	33 *[3]* (3.2 ± nd)	33 *[3]* (5.1 ± nd)	100 *[1]* (4.0 ± nd)	100 *[4]* (5.0 ± 0. 4)	100 *[4]* 2.5 ± 0.4	100 *[4]* (4.8 ± 0.3)	100 *[4]* (2.8 ± 0.5)
**3**		75 *[4]* (4.6 ± 0.8)	67 *[3]* (2.2 ± 1.1)	100 *[6]* (5.4±0.5)	67 *[6]* (2.7 ± 0. 6)	100 *[5]* (4.5 ± 0.1)	100 *[5]* (2.5 ± 1.2)	100 *[5]* (4.7 ± 0.5)	40 *[5]* (3.4 ± 0.5)	100 *[6]* (4.7 ± 0.4)	33 *[6]* (4.2 ± nd)	67 *[3]* (4.2 ± 0.7)	100 *[2]* (2.8 ± 1.5)	100 *[5]* (5.0 ± 0.3)	100 *[5]* (3.7 ± 1.2)	100 *[6]* (4.5 ± 0.1)	50 *[6]* (3.8 ± nd)
**7**		75 *[4]* (4.3 ± 2.0)	67 *[3]* (4.0 ± nd)	50 *[4]* (5.1± nd)	100 *[2]* (2.4 ± nd)	100 *[8]* (4.9 ± 1.0)	100 *[8]* (3.5 ± 1.0)	100 *[5]* (4.9 ± 0.3)	100 *[5]* (3.7 ± 0.4)	80 *[10]* (4.9 ± 0.9)	100 *[8]* (3.1± 1.0)	100 *[3]* (5.0 ± 0.1)	100 *[3]* (3.7 ± 0.8)	100 *[5]* (5.4 ± 0.7)	100 *[5]* (4.4 ± 0.6)	100 *[6]* (4.7 ± 0.4)	100 *[6]* (3.8 ± 0.5)
**12**		83 *[6]* (3.5 ± 1.0)	80 *[5]* 2.4 ± 0.6	100 *[4]* (5.0 ± 0.9)	100 *[4]* (2.4 ± 0.2)	100 *[9]* (4.1± 1.3)	78 *[9]* (3.0 ± 0.9)	100 *[6]* (4.0 ± 0.6)	83 *[6]* (1.7 ± 0.7)	100 *[6]* (5.0 ± 0.2)	100 *[6]* (3.0 ± 0.4)	83 *[6]* (4.3 ± 0.7)	100 *[5]* (2.8 ± 0.8)	100 *[5]* (3.9 ± 0.8)	100 *[5]* (2.4 ± 0.5)	100 *[3]* (3. 9 ± nd)	67 *[3]* (2.9 ± nd)
***Cumulative rate***		**79** *[11/14]*	**73** *[8/11]*	**86** *[12/14]*	**83** *[10/12]*	**100** *[22/22]*	**91** *[20/22]*	**100** *[16/16]*	**75** *[12/16]*	**91** *[20/22]*	**80** *[16/20]*	**83** *[10/12]*	**100** *[10/10]*	**100** *[15/15]*	**100** *[15/15]*	**100** [15/15]	**73** *[11/15]*
**IR** *[+B/tested]*^*c*^	**DR** *[+W&L/+B]*^*d*^																

d.p.e., day post exposure; IR, Infection rate, number of positive bodies/number of tested fed females; DR, Dissemination rate number of positive legs plus wings/number of positive bodies; m.t. ± SD, mean titer ± standard deviation

^a^*[n]*, number of mosquitoes analyzed for the virus presence in the bodies

^b^*[n]*, number of mosquitoes, with positive bodies, analyzed for the virus presence in the legs plus wings

^c^ *[+B/tested]*, cumulative number of positive bodies/tested mosquitoes from 3 to 12 d.p.e.

^d^ *[+W&L/+B]*, cumulative number of positive legs plus wings/positive bodies from 3 to 12 d.p.e.

Regarding the IR, very high values in all dissected mosquitoes were observed at each collection time with the highest number of infected females starting from 3 d.p.e. (values ranging from 67% to 100%). In particular, cumulative IR percentages, calculated as the total number of mosquitoes infected from 3 to 12 d.p.e., were very high in all eight tested populations with values ranging from 79–100% (Table 1). After the 2 d.p.e., disseminated infection was observed for Borgo Vodice, Termoli, Rende, Marina di Zambrone, Cagliari, and Sant’Antioco while, after 3 d.p.e. it was found in Legnaro and Rome with mean viral titers ranging from 3–4 log_10_ PFUeq/mL (Table 1). For all these populations the trend of the mean viral titers were comparable without any statistically significant differences (Kruskal Wallis test *p =* 0.609; *nptrend* values ranging from 0.319 to 0.947).

A value of DR higher than 60% was observed in all specimens collected at 7 d.p.e., with a proportion of the number of mosquitoes CHIKV positive in legs plus wings very high also at 12 d.p.e. (range 67%-100%). Two out of 8 populations (Marina di Zambrone and Cagliari) showed a cumulative DR value of 100%, and high cumulative DR percentages were also obtained from the other populations (range 73–91%) showing a high susceptibility of all populations tested to CHIKV (Table 1)

### Presence of infectious CHIKV in the saliva

In order to determine the length of viral EIP, known to be short in *Ae*. *albopictus* infected with CHIKV E1-226V variant, three populations representative of Central, Southern, and Island Italy, Borgo Vodice, Rende, and Sant’Antioco respectively, were monitored until 12 d.p.e. and saliva was analyzed. Even if the number of positive saliva was low and some of them were at the limit of detection, a viral trend similar to that obtained in bodies and legs plus wings (Table 1) was observed over time. In addition, the viral titers trend in the saliva showed no significant differences among the three populations analyzed (*nptrend* values ranging from 0.49 to 0.82).

**Fig 2:**
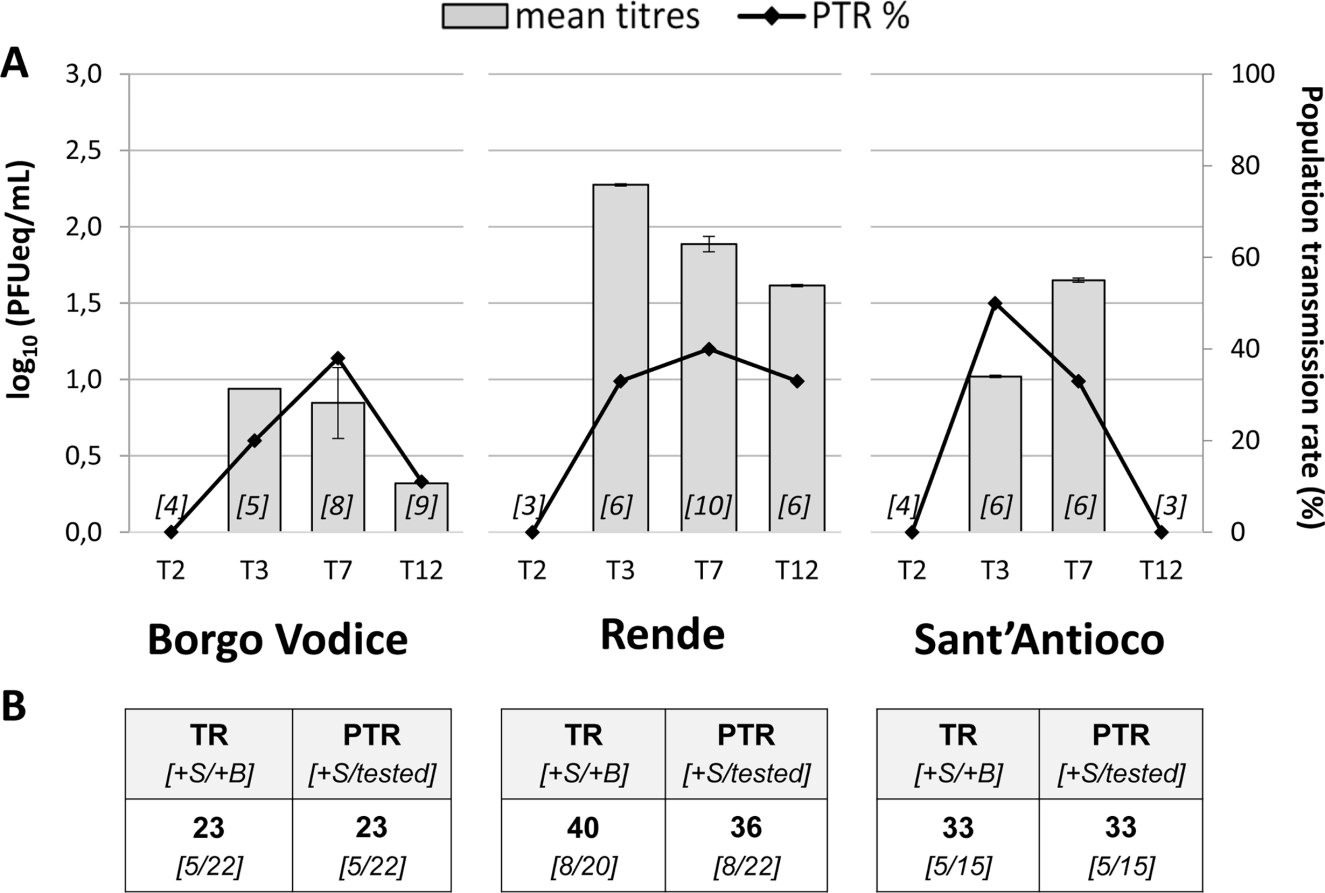
Transmission of CHIKV (E1-226V) in *Aedes albopictus* populations from Central, Southern, and Island Italy. (A) Mosquito saliva samples were collected at 0, 2, 3, 7, and 12 days post exposure (d.p.e.) to an infectious CHIKV blood meal. Population transmission rate (PTR) and the viral mean titer, calculated by qRT-PCR and expressed in PFUeq/mL, were analyzed for all collection time; the number within the grey columns (in square bracket) represent the number of tested mosquitoes analyzed for the presence of the virus in the saliva. (B) The cumulative transmission rate (TR) and PTR of analyzed *Ae*. *albopictus* populations were calculated from 3 to 12 d.p.e. TR corresponding to the proportion of mosquitoes with positive saliva with respect to the number of mosquitoes with positive body *[+S/+B]*; PTR corresponding to the proportion of mosquitoes with positive saliva among the total number of tested fed mosquitoes *[+S/tested]*.

As expected the EIP was very short and viral presence was detected at 3 d.p.e. with mean viral titer of 0.9 log_10_ PFUeq/mL for Borgo Vodice, 2.3 log_10_ PFUeq/mL for Rende and 1.0 log_10_ PFUeq/mL for Sant’Antioco (Fig 2). The maximum value was reached between 3 and 7 d.p.e. in Rende population showing the higher viral titer (2.3 log_10_ PFUeq/mL) at day 3. In accordance with the viral trend observed in bodies and legs plus wings (Table 1), the viral presence decreased to 0.3 log_10_ PFUeq/mL and 1.6 log_10_ PFUeq/mL for Borgo Vodice and Rende, respectively and it was undetectable for Sant’Antioco at 12 d.p.e.

The highest PTR value was recorded at 3 d.p.e. in Sant’Antioco population (50%), while at 7 d.p.e Borgo Vodice (38%) and Rende (40%) were found to have the higher number of mosquitoes with positive saliva of those tested (Fig 2). Moreover, all saliva samples of the three populations tested induced CPE when seeded on VERO cells, confirming the presence of viable CHIKV in these samples.

In Fig 2, cumulative TR and PTR were also showed. Out of the total of infected mosquitoes, 23% of Borgo Vodice, 40% of Rende, and 31% of Sant’Antioco were able to secrete CHIKV by the saliva. Even if the highest cumulative TR and PTR values were recorded in Rende and Sant’Antioco populations, no statistically significant differences in percentages were observed among the tested populations (*p* = 0.51 and *p* = 0.52, respectively). Despite the high variability of CHIKV viral titers in the saliva and the low number of mosquitoes with positive saliva, these findings clearly demonstrate that in all three *Ae*. *albopictus* populations tested, CHIKV is able not only to infect and disseminate very efficiently, but also to reach the salivary glands. It must be taken into account that 2 out of 3 of the mosquito populations (Rende and Sant’Antioco) showed CHIKV positivity in their saliva at 12 d.p.e. with detectable value of 1.6 log_10_ PFUeq/mL in Rende (Fig 2).

### Offspring analysis

In order to detect a possible vertical transmission of CHIKV, adult specimens from the FGC (182 females and 204 males) of the mosquitoes of the eight *Ae*. *albopictus* populations, exposed to the infected blood meal, were analyzed in pools. Two pools (8 females and 37 males) of the Borgo Vodice population were also processed for the SGC. No evidence of vertical transmission was detected in both FGC and SGC progeny.

## Discussion

*Aedes albopictus*, introduced in Italy since 1990, is currently established and has spread throughout the country, especially in urban areas, where it can reach very high densities in the hot season [42, 43].

The CHIKV outbreak in 2007 showed how Europe is vulnerable to the transmission of tropical arboviruses; thus, the risk of new clusters of local transmission cannot be considered negligible for this virus as well as for other imported viruses such as dengue and Zika [44]. It should be considered that repeated CHIKV introductions, occurring through viremic travellers returning from endemic areas, are the main cause of the onset of past and recent epidemic outbreaks, in particular if environmental and/or climatic conditions are suitable for *Ae*. *albopictus* activity. Overall, between 2014 and 2016, a total of 128 imported cases of CHIK infection were notified in Italy; most of them with a travel history to Central and South America [34].

In our work, we initially assessed the susceptibility to infection and dissemination of CHIKV in eight Italian *Ae*. *albopictus* populations representative of the whole country. It should be taken into consideration that the probably different origin of these Italian mosquito populations, introduced in Italy separately from the different tropical and subtropical areas in the last three decades [43, 45], could be the basis of a possible difference in the vector competence. Our results have shown that all eight populations have the same ability to be infected and to disseminate the virus with mean viral titers comparable to each other (no statistically significant differences were reported). Indeed, all the studied populations, exposed to an infectious meal with a final virus concentration which in previous literature showed to induce a high disseminated infection [20, 21] (6.3 log_10_ PFUeq/mL), were found to have high cumulative IR values, ≥ 79%, with high dissemination rates, reaching 100% in 5 of them, already on 2 d.p.e. Furthermore, for the Northern populations our data on susceptibility have also been compared with those previously reported in the literature. In fact, *Ae*. *albopictus* from Northern Italy was already known to be an efficient vector for the CHIKV-E1-226V variant, as confirmed by its involvement in the CHIK outbreak which occurred in Emilia Romagna in 2007 [10, 28, 29]. Therefore, we have focused our study on the transmission of CHIKV in *Ae*. *albopictus* populations originating from Central, Southern, and Island Italy (Borgo Vodice, Rende and Sant'Antioco respectively) analyzing their saliva. This was the first study on EIP of CHIKV in populations originating from these areas.

In the three populations analyzed, also for transmission capacity, virus particles were detected in the saliva starting from 3 d.p.e. confirming a short EIP also for *Ae*. *albopictus* collected in Central, Southern, and Island Italy. It is well known that the EIP of CHIKV in *Ae*. *albopictus* is expected to be below 10 days, usually 5 to 8 days under experimental conditions, and it can be shortened at higher temperatures, in natural conditions, and in particular, when the mutated ECSA E1-226V strain is involved [12, 20, 28, 46]. Indeed, the CHIKV-E1-226V variant presents high levels of replication in *Ae*. *albopictus* that enable the virus to be found early in the saliva [20, 28, 30]. The viral titers trend in the saliva showed no significant differences among the three populations even if the low number of specimens and the low titers value obtained may have influenced this analysis. However, it should be taken into account that on the whole the low values titer of CHIKV RNA detected in saliva specimens of Borgo Vodice, Rende, and Sant’Antioco populations (range: 0.3–2.3 log_10_ PFUeq/mL) are comparable to those found in other similar studies [28, 31, 32, 47]. Indeed, given that it is not possible to provide any control over the salivation, we hypothesize that the high variability of CHIK viral titers in the saliva of infected females at each collection time, could depend on small amounts of saliva produced or/and the limited number of available specimens analyzed. Moreover, experimental conditions could also affect the salivation in this mosquito species, as described by Dubrulle et al. 2009 [30]. It is interesting to note that two out of three populations analyzed (Rende and Sant’Antioco) showed CHIKV positivity in their saliva up to the 12 d.p.e. with detectable values (1.6 log_10_ PFUeq/mL) suggesting a possible risk of viral transmission later during the infection process. These mosquito populations not only became competent transmitters of the viable virus in a short time, but they can also maintained a persistent infectious virus in the mosquitoes over time. *Ae*. *albopictus* with persistently infected salivary glands, may represent a concern due to their high epidemic potential. Since it is known that viral replication is strongly conditioned by intrinsic factors of the mosquito that can modulate the virus's ability to escape from midgut or to reach and infect the salivary glands [48–50], could be of interest to identify which vector intrinsic factors are involved in the tropism of CHIKV E1-226V in the Italian *Ae*. *albopictus* tested.

The cumulative TRs and PTRs of the three selected populations, with values ranging from 23–40% and 23–36% respectively, showed a comparable vector competence among these Italian *Ae*. *albopictus* populations. The high values of TR obtained between 3 and 7 d.p.e. were consistent with findings in previous studies where *Ae*. *albopictus* was shown to be very efficient in the transmission of CHIKV E1-226V with a delivery in the saliva at 3 d.p.e. [28, 30, 32].

Mosquito adults from the FGC of the eight population (N = 386) and from the SGC of Borgo Vodice colony (N = 45) were analyzed to detect the virus in the progeny. We did not find any evidence of viral particles in the pools. However, even if the number of specimens processed was limited, in particular in the SGC, our findings were consistent with the results of previous studies, supporting the hypothesis that vertical transmission of CHIKV represents a rare event in *Ae*. *albopictus* populations, either in experimental infections or in field conditions in endemic areas [20, 48, 51–54].

In summary, our experimental findings confirm that *Ae*. *albopictus* represents a potential threat in Italy, playing an important role as a vector of CHIKV, in particular where high densities of the species are seasonally recorded [43]. The recent CHIKV outbreaks in South-Eastern France and Central-Southern Italy [15, 16, 33] testify to this concern, and the risk of local transmission of imported arbovirus from viremic travellers returning from affected areas of the world has become a reality for Europe [10, 13–16, 34, 44].

### Conclusion

Understanding virus-vector interactions remains essential for risk assessment, and additional studies to evaluate differences in vector competence of *Ae*. *albopictus* to different CHIKV strains are needed for epidemic preparedness. Moreover, in absence of vaccine and/or specific treatment active surveillance has to be considered the most important approach to control CHIKV outbreaks for providing early warning and for applying appropriate vector control strategies.
